# Biochemistry of Heat Shock Proteins From Human Intracellular Protozoan Parasites as Diagnostic and Therapeutic Biomarkers

**DOI:** 10.1021/acs.biochem.5c00120

**Published:** 2025-06-02

**Authors:** Graham Chakafana, Tawanda Zininga

**Affiliations:** 1 Department of Chemistry and Biochemistry, 3726 Hampton University, Hampton, Virginia 23668, United States; 2 Department of Biochemistry, 26697 Stellenbosch University, Stellenbosch 7600, South Africa

**Keywords:** human parasite, heat shock protein, biomarker, drug targets, diagnostic marker

## Abstract

The main protozoan parasites, including Plasmodium, Leishmania, Toxoplasma, and Trypanosoma, face significant environmental stress during their life cycles. To survive, they rely on heat shock proteins (Hsps), which play essential roles in protein folding, preventing aggregation, and stabilizing cellular pathways under stress. Due to their critical functions, parasite Hsps have emerged as promising drug targets and potential diagnostic biomarkers. Several studies have revealed structural and functional differences between parasite and human Hsps, making them attractive for selective drug targeting. However, challenges such as specificity and host toxicity remain obstacles in Hsp-targeted therapies. Additionally, several key questions remain unanswered: What unique adaptations allow parasite Hsps to function efficiently? How do they interact with other chaperone systems? What roles do they play in parasite virulence and host–pathogen interactions? Addressing these gaps will enhance our understanding of parasite biology and support the development of more effective therapeutic and diagnostic strategies. This review evaluates the current knowledge on parasite Hsps, their potential as drug targets, and approaches to overcome existing challenges. Gaining deeper insights into their mechanistic roles could lead to safer and more targeted interventions against protozoan infections.

## Graphical abstract

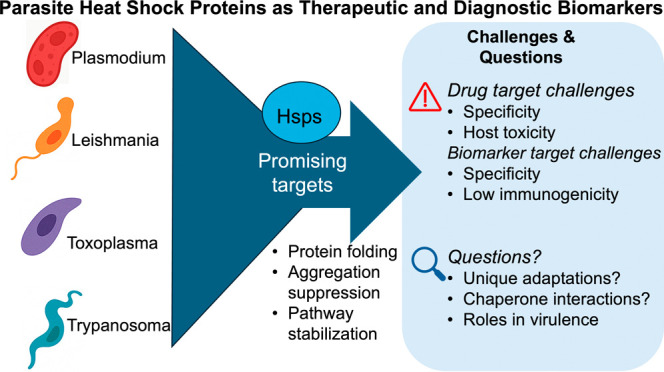

## Introduction

The main protozoan parasites that infect humans are from Plasmodium, Toxoplasma, Trypanosoma, and Leishmania species (Table 1). They cause malaria, toxoplasmosis, chagas disease, and leishmaniasis, respectively.^1^ Together, these parasites cause an estimated 1 million deaths annually, mostly in the temperate regions of the tropics.^2^ Among these, malaria accounts for the most serious disease, and the others are grouped as neglected tropical diseases. These parasites have a common stage in their lifecycles that thrives by hijacking the human system to reproduce and multiply.^3^ This review explores the questions on the biochemical logic of targeting the parasite protein folding system, mainly heat shock proteins (Hsps), as therapeutic and diagnostic biomarkers. These proteins exhibit distinct chemical structures and biochemical functions that facilitate protein folding, ultimately promoting parasite survival. However, these same unique properties also present vulnerabilities that could be exploited for therapeutic intervention. The key question, therefore, is which structural and functional features of parasite Hsps can be selectively targeted using biochemical countermeasures to induce parasite death?

**Table 1:** Characteristics of Human Obligate Intracellular Parasites

**parasite and species**	**disease**	**transmission**	**target host cell**	**comments**
Plasmodium *P. falciparum*, *P*. *vivax*, *P. ovale*, *P. malariae*, *P. knowlesi*	malaria	through *anopheles* mosquito bite	red blood cells and liver cells	invades red blood cells to escape immune detection and exploit host nutrients for replication
*Toxoplasma gondii*	toxoplasmosis	ingestion of oocyst in contaminated food/soil/cat litter	macrophages and various nucleated cells	forms intracellular cysts, allowing it to persist in host tissues and evade immune responses
*Leishmania spp*	Leishmaniasis: cutaneous	bite from infected *Phlebotomine* sand flies	macrophages	live in phagolysosomes of phagocytes, adapting to acidic conditions and avoiding immune clearance
*L. major*, *L. tropica*, *L. mexiacana*,*L. amazoniensis*,*L. braziliensis*,*L. guyanensis*,L. panamensis, *L. peruviana*	mucocutaneous			
*L. braziliensis*	visceral			
*L. panamensis*, *L. guyanensis*				
L. donovani, L. infantum (L. chagas)				
*Trypanosoma cruzi*	Chagas disease	triatomine bug (kissing bug)	muscle cells, neurons, macrophages, fibroblasts	form amastigotes in host cells to hide from immune surveillance and to multiply
T. brucei gambiense and *T. rhodesiense*	African trypanosomiasis sleeping sickness	tsetse fly (Glossina)		

### Understanding Heat Shock Protein Biological Activity in Parasites

Hsps are molecular chaperones that noncovalently assist clients to fold and achieve their three-dimensional functional structure. Generally, Hsps are also involved in the suppression of protein unfolding, aggregation, translocation, and channeling the misfolded proteins for proteolysis to maintain protein homeostasis. Hsps are ubiquitous as they are found in all cellular organisms and exhibit a high level of conservation, which highlights their important role in cell survival. These proteins are grouped based on their molecular sizes and functional roles (Figure 1). Most of our current understanding of the function of Hsps is from the bacterial E. coli systems. There is, however, an increase in the number of reports on the role of Hsps in parasites, which has mostly been on P. falciparum, L. major and L. donovani species; the other protozoan parasites have not received enough attention (Table 2).

**Figure 1:**
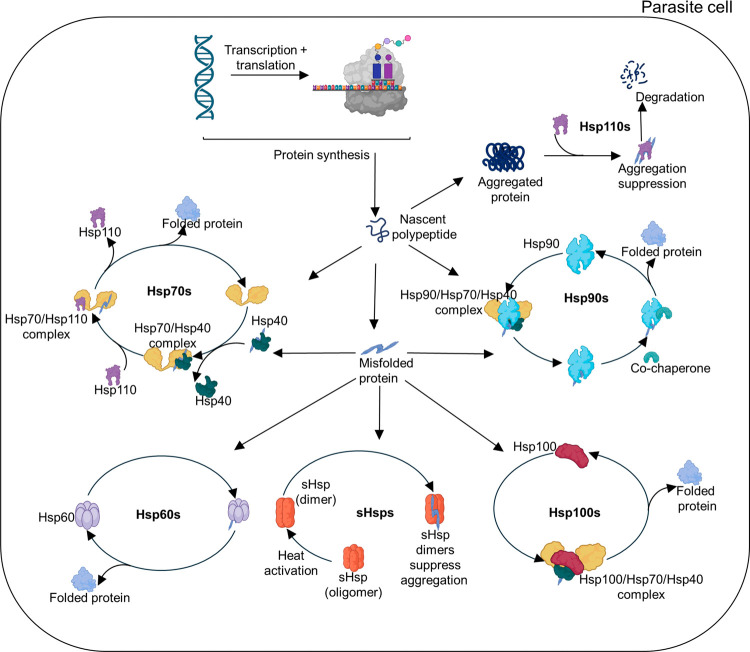
Role of chaperones in cellular proteostasis and their functional families. Nascent polypeptides emerging from ribosomes may spontaneously fold into their native conformation; however, many require assistance from Hsps to achieve proper folding. Among the first chaperones to interact with these polypeptides are Hsp40/JDPs, which facilitate substrate recruitment to Hsp70 for initial folding. Partially folded polypeptides may subsequently be transferred to Hsp60 or Hsp90 for further maturation and activation. In cases where misfolded proteins aggregate, Hsp100, in collaboration with the Hsp40/Hsp70/Hsp110 system, aids in resolubilizing these aggregates. In parasitic organisms, sHsps are thought to play a crucial role in preventing protein aggregation. Although they lack intrinsic folding activity, sHsps bind unfolded proteins and transfer them to ATP-dependent chaperones for proper refolding and stabilization.

**Table 2:** Summary of the Main Parasite Heat Shock Proteins

**protein family**	**species and number of genes**	**localization**	**stress function**	**references**
Hsp110	*P. falciparum* - 2	cytosol	nucleotide exchange factor, thermotolerance, holdase activity	^4^
		ER		^5^
	*L. major* - 4	?		
	*T. gondii* -	?		
	*T. cruzi* - 4	?		^6^
Hsp100	*P. falciparum* - 8	mitochondria, apicoplast, parasitophorous vacuole	disaggregase, ATPase proteolytic, protein export	^7–9^
	*L. major* - 4	cytosol	confers thermotolerance during the development from the promastigote to the amastigote stage.	^10,11^
			ATP-dependent unfoldase that promotes protein disaggregation or facilitates the unfolding of aggregation-prone polypeptides marked for degradation.	
	*T. gondii* - 10	cytosol	protein refolding and repair	^12^
	*T. cruzi* - 3	cytosol	refolding misfolded proteins, preventing protein aggregation, and maintaining proper protein function.	^13^
Hsp90	*P. falciparum* - 4	cytosol, mitochondria	ATPase, foldase, associates with Hsp70, kinases	^14–16^
		ER		
		apicoplast		
	*L. major* - 18	cytosol, ER, Nucleus	protein folding, stabilization, and maturation of proteins involved in parasite survival	^17^
	*T. gondii* - 4	cytosol	assist in the proper folding and maturation of newly synthesized proteins, ensuring they are functional and preventing protein aggregation, which is critical for the parasite’s survival in the host environment.	^18,19^
			Hsp90 is also involved in the transition from the tachyzoite stage to the bradyzoite stage in the parasite’s life cycle.	
	*T. cruzi* - 4	cytosol	protein folding, activation, disaggregation, prevention of aggregation	^20,21^
Hsp70	*P. falciparum* - 4	Cytosol	ATPase, foldase, interacts with Hsp40 and Hsp90, requires nucleotide exchange factors GrpE/Hsp110/Bag.	^22–26^ .
		ER, mitochondria		
		exported to RBC		
	*L. major* - 9	cytosol, mitochondria, ER	protein folding, aggregating suppression, facilitates the transport of proteins across the endoplasmic reticulum and mitochondria.	^27^
	*T. gondii* - 5	cytosol	facilitates protein folding, assembly, and transport.	^28^
			plays a key role in the transition between the tachyzoite and bradyzoite stages of the life cycle.	
	*T. cruzi* - 8	cytosol, nucleus	protein folding, transport, and preventing protein aggregation	^29,30^
Hsp60	*P. falciparum* - 2	mitochondria and cytosol	ATPase, foldase, hydrolysis activated by Hsp10	^31^
	*L. major* - 4	mitochondria, cytoplasm, ER	ensures proper folding of polypeptides, prevents aggregation of misfolded proteins, and assists in their refolding to maintain cellular proteostasis.	^32^
	*T. gondii* - 10	mitochondria and cytosol	ensures proper protein folding and structural integrity within the parasite’s mitochondria, supporting survival and differentiation throughout its life cycle.	^33^
	*T. cruzi* - 6	mitochondria	facilitates the folding and assembly of newly synthesized proteins, especially during critical life cycle transitions of the parasite.	^34^
Hsp40	*P. falciparum* - 49	cytosol	cochaperone of Hsp70, J-dots, protein export	^35–37^
		ER		
		apicoplast, RBC cytosol exported		
	*L. major* - 69	cytosol, ER	assists Hsp70 in protein folding, unfolding, and transport by binding to unfolded or misfolded proteins, delivering them for proper folding, and preventing aggregation within the parasite.	^38,39^
	*T. gondii* - 36	cytosol, ER	Hsp70 cochaperone that facilitates protein folding.	^40^
	*T. cruzi* - 73	cytosol, ER	Hsp70 cochaperone that assists in protein folding, prevents aggregation helping the parasite survive under stressful conditions throughout its complex life cycle.	^41^
small Hsps	*P. falciparum* - 3	mitochondria, cytosol, ER	holdase cochaperone of Hsp70/Hsp90	^42^
	*L. major* - 3	cytosol, ER	maintains protein solubility and prevents misfolded protein accumulation.	^39^
			likely contributes to maintaining membrane stability and integrity.	
	*T. gondii* - 4	cytosol, mitochondria	ensures proper protein folding and prevents the accumulation of misfolded proteins.	^43^
	*T. cruzi* - 2	cytosol, mitochondria		^13^

### Hsp100 Family

Hsp100 proteins play crucial roles in the untangling of aggregated protein complexes, also known as disaggregases, and are required for thermotolerance.^44^ In parasites, the mechanisms of action of these proteins are not well understood, but there is growing evidence from structural studies on P. falciparum and *Leishmania* spp. homologues. The Leishmania major Hsp104 has been shown to be upregulated under heat stress and is involved in thermotolerance.^11^ It was shown to be important during the amastigote differentiation and virulence.^10^ The differential expression pattern of the Hsp100 in L. majorwas proposed to present a unique selective targeting of Hsp100s as it has been linked to exosome biogenesis, influencing the protein cargo composition of extracellular vesicles, which play roles in immune modulation and pathogenesis.^45^ Moreover, it has been proposed that the N terminus NBD of L. major Hsp100 exhibits substrate binding capability and is rich in methionines and aromatic amino acids, which potentially facilitate the protection of the parasite from oxidative damage in the macrophages.^46,47^ This, in part, shows species-specific adaptation of Hsp100 for parasite survival. In P. falciparum, the Hsp100 ortholog, known as Hsp101, is essential for the parasite’s development within erythrocytes. *Pf*Hsp101 is involved in the formation of the parasitophorous vacuole translocon parasite protein export complex (PTEX) involved in the export of virulence factors into the host cell.^48^ Notably, *Pf*Hsp101 possesses unique sequence insertions not found in human homologues, presenting a potential target for antimalarial drug development. The T. gondii Hsp100 was reported to function similarly to other apicomplexan parasites like plasmodium in thermotolerance and aiding in protein quality control during stress-induced differentiation processes.^49^ Lastly, in Trypanosoma, mainly in *T. cruzi* and T. brucei, Hsp100 contributes to the parasite’s ability to cope with oxidative and heat stress encountered during infection.^50,51^ The protein assists in the refolding of oxidatively damaged proteins, thereby maintaining cellular integrity. This function is particularly important during the parasite’s transition between insect vectors and mammalian hosts. Taken together, Hsp100s play important roles in the survival of protozoan parasites, aiding in protein disaggregation, stress response, and life cycle progression. While sharing common functions, parasite Hsp100s possess unique structural and functional features that distinguish them from their bacterial and human counterparts. These distinctions provide valuable insights into the development of targeted therapies against parasitic infections.

### Hsp90 Family

Heat shock protein 90s (Hsp90) are considered secondary ATP-dependent molecular chaperones as they assist the folding, stabilization, and activation of nearly folded clients. Most of the clients of Hsp90 include kinases and transcription factors essential for cell cycle progress and signal transduction. Hsp90 is implicated in regulating critical life cycle transitions in these parasites. For instance, in P. falciparum, the cytosolic Hsp90 modulates the transition from ring to trophozoite stages during asexual development within human erythrocytes and is upregulated under stress conditions.^14,52^ Similarly, in Leishmania species, Hsp90 is involved in the differentiation from promastigote to amastigote forms, a process essential for establishing infection in mammalian hosts.^53–55^ It is interesting to note that the Leishmania spp have an expanded Hsp90 family (Table 2), suggesting specialized adaptations to the parasite’s intracellular environment.^17^ However, it remains to be validated why there is this expanded family and what evolutionary advantages does this present to the parasite.

Despite similarities among Hsp90s, there are species-specific differences between parasite Hsp90s. The cytosolic P. falciparumHsp90 (*Pf*Hsp90) exhibits approximately 40% sequence divergence from human Hsp90, with a notable 30 amino acid insertion in the linker region adjacent to the ATP-binding domain.^56^ This insertion influences PfHsp90s ATPase activity and its interaction with specific inhibitors, such as geldanamycin and other accessory proteins.^57–59^ In Leishmania species, Hsp90 is essential for the parasite’s survival under stress conditions encountered within the host. The Hsp90 heterocomplex in Leishmania includes unique cochaperones that are not present in mammalian systems.^17,60^ In T. gondii, Hsp90 expression is upregulated during stress-induced differentiation from tachyzoite to bradyzoite forms.^61^ Experimental disruptions of Hsp90 expression impair the parasite’s ability to invade host cells and undergo stage differentiation, underscoring its critical role in T. gondiipathogenicity.^18,62^ Trypanosoma species exploit Hsp90 to facilitate their complex life cycle transitions between insect vectors and mammalian hosts. Similarly to Hsp40s, Hsp90 in these parasites is involved in the stabilization of variant surface glycoproteins, which are essential for immune evasion.^63^ The unique client protein repertoire of trypanosomal Hsp90 distinguishes it from its counterparts in other organisms.

It is interesting to note that among the parasite Hsps, Hsp90 is the most targeted family in drug screening, but there is still limited understanding of its function in the parasites. One of the primary obstacles to advancing comprehensive studies of parasite Hsp90s has been the technical difficulty in producing their recombinant full-length forms. In P. falciparum, for instance, the expression of functionally active, full-length Hsp90 using conventional heterologous systems such as E. colihas proven particularly challenging due to issues like protein misfolding, aggregation, and the absence of post-translational modifications.^64^ As a result, functional characterization and structural studies on inhibition have predominantly relied on truncated subdomains of Hsp90, which, although more tractable, fail to capture the complexity of the full-length protein.^65–70^ Nonetheless, significant strides have recently been made in overcoming these expression challenges. Optimized expression systems, particularly those using E. coli, have enabled the successful production of full-length parasite Hsp90s with improved solubility and functional activity.^16,56,69,71^ These advances mark a pivotal shift in the field, opening new possibilities for high-throughput screening and mechanistic studies. Moreover, the integration of rational drug design strategies, enhanced by artificial intelligence (AI) and machine learning algorithms, is promising to accelerate the identification of selective Hsp90 inhibitors tailored to parasitic systems. Such multidisciplinary approaches, as highlighted in recent reviews,^72^ hold substantial promise for unlocking the therapeutic potential of parasite Hsp90s, moving beyond the limitations imposed by earlier methods and conceptual frameworks.

### Hsp60 Family

Heat shock protein 60 (Hsp60), also known as chaperonin 60 (Cpn60), is a highly conserved molecular chaperone that plays a critical role in protein folding, assembly, and quality control in various organisms, including parasites. In these parasitic protozoa, Hsp60 is essential for cellular survival, particularly under stress conditions encountered during host infection.^33,34,73,74^ Despite its conserved function, parasite Hsp60 exhibits species-specific adaptations that contribute to the pathogenicity and survival in different hosts. Hsp60 belongs to the chaperonin family and functions as a double-ring oligomeric complex that encapsulates unfolded proteins in an ATP-dependent manner.^75^ The ATP hydrolysis cycle drives conformational changes necessary for substrate binding and folding, a mechanism conserved across the protozoan parasites.^76^ Like its eukaryotic homologue Hsp60 in mammals, parasite Hsp60 is predominantly localized in the mitochondria, where it assists in folding mitochondrial proteins.^77^ In all four parasites, Hsp60 ensures the stability of mitochondrial proteins under oxidative stress, a common feature of host–pathogen interactions.^33,73,74,78^ Parasite Hsp60 is upregulated in response to heat stress, oxidative stress, and drug pressure, suggesting a conserved role in stress adaptation.^3^ In all species, Hsp60 interacts with cochaperones such as Hsp10, forming a functional chaperonin complex that assists in refolding stress-damaged proteins.^79^

There is a paucity of data on the function of Hsp60s in parasites, but most studies are based on the bacterial Hsp60, GroEL the bacterial system. In Apicomplexan parasites, P. falciparumand T. gondii, Hsp60 is dually localized to the mitochondria and apicoplast, two essential organelles required for parasite survival and development.^33,80^ Within the apicoplast, Hsp60 assists in protein folding, maintaining apicoplast proteostasis and metabolic functions such as fatty acid and isoprenoid biosynthesis.^81,82^ In T. gondii, Hsp60 appears to have additional roles beyond proteostasis, particularly in regulating the parasite’s developmental switch between tachyzoite and bradyzoite forms, a transition central to chronic infection and cyst formation.^33^ Since the apicoplast is absent in human cells, future studies exploring substrate specificity and expression dynamics in apicoplast Hsp60 may guide the design of novel inhibitors with selectivity for this organellar chaperone. *Leishmania* spp. Hsp60 is also found to be a common parasite antigen, where it plays a role in host immune evasion.^32^ It is plausible that surface-expressed Hsp60 interacts with host macrophages, modulating inflammatory responses and contributing to parasite survival within phagolysosomes. This immunomodulatory function is more prominent in *Leishmania* than in other parasites. On the other hand, T. bruceiHsp60 is implicated in the folding of variant surface glycoproteins (VSGs), which are crucial for antigenic variation and immune evasion.^83,84^ This role is unique to Trypanosomes, as antigenic variation is a major survival strategy in these parasites.

### Hsp40 Family

Heat shock protein 40 (Hsp40) family members are also known as J-domain proteins (JDP) based on the presence of a conserved J-domain that mediates the interaction with Hsp70s, stimulating their ATPase activity. In parasitic protozoa, the JDP family is expanded and diverse, reflecting the need of the parasite to adapt to varying host environments (Table 2). In the malaria parasites, there is a repertoire of Hsp40s that are implicated in the trafficking of virulence factors to the host RBC surface, thereby contributing to immune evasion and pathogenicity.^36,85,86^ Similarly, in T. gondii, JDPs are thought to be involved in assisting protein folding during the parasite invasion and replication within host cells.^40^ However, in Leishmania, JDPs are also involved in differentiation processes between promastigote and amastigote forms, aiding parasite survival in a hostile host macrophage environment.^38^ On the other hand, in Trypanosoma spp. these proteins are involved in maintaining the parasite mitochondrial DNA.^87^ Since these are the expanded family of Hsps across parasitic species, there is limited understanding of the precise roles of respective members in parasite biology and pathogenicity. The networks of JDP interactions with Hsp70 and other cochaperones in parasites are not well-defined. Mapping these interactions is crucial for comprehending how protein folding and stress responses are coordinated. Considering that some JDP members have a C-terminus substrate binding domain, which exhibits independent substrate binding activity and suppression of substrate aggregation.^88^ These are thought to recruit these substrate and bring it to Hsp70. However, for the members without a substrate binding capability, how do they modulate substrate folding? Do they solely assist Hsp70 recruitment to the substrate site, such as the j-dots described in P. falciparumby Külzer and colleagues?^89^

### Hsp70 Family

Hsp70 is a ubiquitous molecule capable of both suppressing protein aggregation and refolding misfolded peptides. In parasitic protozoa, Hsp70 exhibits both conserved functions and unique adaptations that are essential for their survival and pathogenicity. Hsp70 assists in the proper folding of nascent and stress-denatured proteins, preventing aggregation and maintaining cellular proteostasis. This function is analogous to Hsp70's role in other organisms, ensuring that proteins attain and maintain their functional conformations. Parasite Hsp70s interact with cochaperones, such as Hsp40, to form functional chaperone complexes.^35,38,41,90,91^ These interactions are vital for regulating Hsp70 ATPase activity and substrate specificity, facilitating efficient protein folding and trafficking within the parasite cells. Exposure to environmental stresses, such as elevated temperatures or oxidative stress, leads to the upregulation of Hsp70 expression in these parasites. This adaptive response enhances the parasites’ ability to survive hostile conditions encountered during transmission and infection.

In P. falciparum, the causative agent of malaria, Hsp70 members are localized not only in the cytosol, ER, and mitochondria but also in the parasitophorous vacuole, the infected Rbc cytosol, and on the surface of infected erythrocytes.^79^ This unique distribution is associated with the trafficking of virulence factors, such as P. falciparumErythrocyte Membrane Protein-1 (PfEMP1), to the host cell surface, contributing to immune evasion and pathogenicity.^92–94^ Additionally, *Pf*Hsp70–1 has been shown to interact with phosphatidylinositol 3-phosphate (PI(3)­P), stabilizing the digestive vacuole under heat stress conditions, which is crucial for parasite survival during febrile episodes.^95^ On the other hand, Leishmania parasites possess multiple Hsp70 isoforms, with Hsp70-II being essential for their survival.^96^ Studies have shown that promastigotes lacking the Hsp70-II gene exhibit significant cellular and biochemical alterations, underscoring its critical role in maintaining parasite viability and infectivity.^97^ In T. gondii, Hsp70 is involved in the parasite’s stress response mechanisms, aiding in the maintenance of protein homeostasis under adverse conditions.^28^ While specific unique features of Toxoplasma Hsp70 compared to other parasites are less well-characterized, its role in stress adaptation is crucial for the parasite’s survival and pathogenicity.^98^ In T. cruzi, Hsp70 is implicated in the parasite’s response to environmental stressors. However, unlike in Leishmania, Hsp70 in T. cruzi and T. brucei is not directly associated with stage differentiation but plays a role in managing stress conditions encountered during the parasite’s life cycle.^6,39^ Hsp70 proteins in these organisms share fundamental roles in protein folding and stress response. However, each parasite has evolved unique adaptations of Hsp70 to meet specific challenges within its respective hosts. These unique features not only underscore the versatility of Hsp70 functions but also highlight potential targets for therapeutic intervention against these parasitic infections.

### Small Heat Shock Proteins

Small heat shock protein (sHsps) family members are 20 kDa in size and are defined by a conserved α-Crystallin domain (ACD). The ACD consists of approximately 80–100 amino acids flanked by variable N-terminal and C-terminal regions. The ACD forms the core of the sHsp structure, facilitating the dimerization and oligomerization that are essential for function. This ACD facilitates the formation of oligomeric complexes, enabling sHsps to act as ATP-independent chaperones that prevent the aggregation of unfolded proteins during cellular stress. This energy-saving property could be advantageous for parasites such as *Plasmodium*, *Trypanosoma*, and *Leishmania*, which often encounter resource-constrained environments during infection. By acting as ATP-independent chaperones, sHsps serve as energy-efficient buffers against proteotoxic stress, helping to stabilize unfolded or misfolded proteins under conditions in which ATP-dependent chaperone systems may be less effective.

There is limited experimental validation of the function of members of this family of chaperones across these parasites, apart from the bioinformatics data set.^99^ However, in protozoan parasites, sHsps are thought to be upregulated in response to environmental challenges such as heat shock, oxidative stress, and changes in pH, reflecting their role in stress adaptation.^100^ In L. donovani, and T. cruzi, Hsp23 is essential for thermotolerance and is upregulated during stress.^101,102^ *Ld*Hsp23 localizes predominantly in the perinuclear region and is crucial for the parasite’s survival at elevated temperatures encountered during transmission from the sandfly vector to the mammalian host. Loss of LdHsp23 results in increased sensitivity to temperature-induced stress, underscoring its protective function.^101^ Similarly, in T. gondii, four Hsp20s (Hsp20, Hsp21, Hsp28, and Hsp29) were shown to be upregulated in tachyzoites during heat-induced stress to maintain cell integrity.^103^ Although the ACD of sHsp is highly conserved across species, parasites possess unique N- and C-terminal extensions,^42^ which could modulate the functional diversity of parasitic sHsps and serve as entry points for therapeutic selectivity. The malaria parasite expresses sHsps that are not yet experimentally characterized, but they are implicated in the development of thermotolerance, aiding the parasite’s survival during febrile episodes in the host.^42^ These proteins assist in maintaining protein stability and function at fluctuating temperatures. In T. cruzi, sHsps are involved in the parasite’s differentiation process and adaptation to stress conditions, contributing to its ability to withstand hostile environments within the host.

### Substrate Recruitment and Functional Adaptations of Hsps in Parasites

The ability of parasite Hsps to selectively recognize and bind a wide range of substrates is critical for maintaining proteostasis in cells. While Hsps generally bind a range of broad substrates, they also demonstrate a remarkable selectivity for distinct classes of proteins, enabling them to differentiate between newly synthesized polypeptides, stress-denatured proteins, and aggregation-prone intermediates.^104,105^ Substrate selection by Hsps is largely dictated by the folding state and conformational properties of the client protein. Hsp70 preferentially interacts with unfolded or extended polypeptides, preventing premature aggregation and facilitating productive folding cycles.^106^ Hsp90, in contrast, binds partially folded substrates, assisting in the structural maturation and functional activation of client proteins. sHsps act as molecular holdases, stabilizing aggregated proteins to prevent further misfolding and irreversible aggregation.^107^ This hierarchical substrate recognition system ensures that proteins receive appropriate chaperone assistance based on their folding status, allowing for efficient triage among folding, stabilization, and degradation pathways to maintain proteostasis under both physiological and stress conditions.

Substrate recruitment and binding selectivity are governed by a combination of structural features, allosteric regulation, and cochaperone interactions. A key determinant of substrate recognition is the exposure of hydrophobic motifs and peptide segments, which remain buried in correctly folded proteins but become accessible upon stress-induced misfolding. Hsp70 and Hsp40 primarily bind hydrophobic residues, which serve as aggregation-prone signals.^11,108,109^ The substrate binding domain (SBD) of Hsp70s contains hydrophobic pockets that accommodate short, unfolded peptide sequences (∼5–7 amino acids), facilitating transient interactions that promote protein refolding.^110^ Notably, parasite Hsp70 chaperones exhibit a binding preference for substrates that are rich in hydrophobic amino acids.^24,106^ It has recently been shown that additional domains on P. falciparumHsp70s, including the interdomain linker and C-terminal extended GGMP motifs, also dictate Hsp70 substrate selectivity.^26,111^ Similarly, Hsp90 preferentially engages client proteins via hydrophobic interactions, often recognizing structurally disordered regions or partially folded β-strands and helical motifs, which influence binding affinity.^112^ While some Hsps display sequence-dependent affinity, others prioritize structural motifs over specific amino acid compositions, allowing for broader client recognition. Through this interplay of sequence selectivity, structural discrimination, and dynamic allosteric regulation, Hsps efficiently navigate the complex energy landscape of protein folding to maintain cellular proteostasis.

Nucleotides also play significant roles in substrate capture and release. Hsp70s and Hsp90s utilize ATP-regulated conformational changes to cycle between open (low-affinity) and closed (high-affinity) states. In the ATP-bound state, Hsp70 opens the SBD, allowing for substrate capture, while ATP hydrolysis locks the substrate in place, stabilizing the unfolded protein.^113^ In addition to nucleotides, the cooperative action of other Hsp types, such as Hsp40 and Hsp90, and cofactors, such as Hop (Hsp70-Hsp90 organizing protein), further enhances substrate specificity by directing specific client proteins to Hsp70.^114^ JDPs with substrate binding capability recognize nascent or misfolded substrates and transfer them to Hsp70, increasing the substrate recruitment efficiency. This coordinated interplay among Hsps refines substrate recognition and ensures an efficient chaperone function. Post-translational modifications including phosphorylation, acetylation, and ubiquitination also modulate Hsp-substrate interactions by altering substrate conformation or chaperone affinity.^115,116^ In addition, human Hsp70s have been shown to preferentially bind phosphorylated clients.^117^ Given the species-specific differences between host and parasite proteomes, it is intriguing how parasite Hsp70 efficiently recognizes and processes its substrates. Studies utilizing a chimeric Hsp70 composed of E. coliand P. falciparumcomponents (dnaK-PF) demonstrated that the substrate binding domain (SBD) of plasmodial Hsp70 exhibits a preference for substrates of malarial origin.^25,118^ The P. falciparumproteome is characterized by a high proportion (24%) of prion-like repeats, which are highly susceptible to aggregation under thermal stress.^119^ To mimic these characteristics, Asp residues were introduced into Hsp70 model peptide substrates, revealing that malarial Hsp70 preferentially binds to substrates that resemble the malarial proteome.^24,120^ These studies using both a full-length and a peptide substrate showed evidence of selective binding of parasite Hsp to a substrate that mimics its proteome.

### Kinetic and Equilibrium Processes

Hsps generally operate under both kinetic (ATP-driven) and equilibrium (steady-state) mechanisms, enabling a balance between rapid response and long-term protein stability. ATP-driven chaperones like Hsp70 operate in open systems, where substrate interactions are transient and regulated by ATP hydrolysis.^106^ This mechanism enables rapid adaptation to cellular stress, ensuring continuous protein quality control. ATP-driven conformational cycling in Hsp70s facilitates transitions, which modulate the chaperone’s kinetic properties of substrate recognition, folding assistance, and degradation within cellular proteostasis networks.^121,122^ In the ATP-bound state, Hsp70 adopts a low-affinity open conformation, allowing dynamic substrate exchange while ATP hydrolysis facilitates the transition to a closed high-affinity state for peptide binding.^120,123^ The plasmodium Hsp70 was shown to exhibit faster ATP hydrolysis rates as compared to human host and bacterial DnaK.^25,124^ This suggests that the kinetic control in Hsp70 ensures rapid adaptation to cellular stress in parasites by possibly modulating substrate interaction rates. As such, parasite Hsp70s are able to process multiple substrates per minute, facilitating efficient protein folding during heat stress. In contrast, equilibrium-driven chaperones such as sHsps and Hsp110 function in a closed system, where substrates remain bound until specific external signals trigger their release. It has previously been shown that P. falciparumHsp110s stabilize partially folded proteins better than parasite Hsp70–1 by maintaining them in a prolonged bound state, which is independent of the nucleotide state.^5^ This suggests that the parasite Hsp110s could be robust buffers to protein misfolding in stress cells depleted of ATP. However, it is not well established how the binding and release events on these ATP-independent chaperones are regulated in parasites. It is plausible that, similar to sHsps that exist in dynamic equilibrium between oligomeric and monomeric states, this could be a regulation mechanism.^125^

Several studies have demonstrated that the Hsp90 chaperone cycle also operates through a series of kinetically driven conformational transitions, balanced by thermodynamic equilibrium between distinct structural states.^126^ Structural studies also reveal that Hsp90 exists in a dynamic equilibrium between open and closed conformations, modulated by ATP binding, hydrolysis, and cochaperone interactions.^56,69,126,127^ In the apo state, Hsp90 adopts an open conformation, which is thermodynamically favored under nucleotide-free conditions. Upon ATP binding, Hsp90 undergoes a series of kinetically controlled structural rearrangements and assumes a closed state. During this stage, unfolded client proteins are recognized and stabilized by cochaperones, facilitating their recruitment onto Hsp90 ^128,129^ ATP hydrolysis serves as a key kinetic checkpoint, triggering the release of substrate and restoring the initial equilibrium state. In parasite cells thriving under stress such as P. falciparum, the Hsp90 has faster ATP hydrolysis cycles than the host Hsp90.^130^ The Hsp90 cycle is thus governed by a unique finely tuned balance between kinetic and equilibrium processes, where ATP binding and hydrolysis provide the kinetic driving force, while interdomain interactions and cochaperone dynamics regulate equilibrium states. This interplay ensures efficient species-selective client processing, allowing Hsp90 to function as a versatile molecular chaperone under varying cellular conditions to which the parasite is exposed in the host.

### Concentrations of Hsps and Substrate Stoichiometry

The function of parasite Hsps is dependent on their concentration relative to substrate proteins, which varies across different cellular conditions and stress responses. As such, understanding the stoichiometric balance between Hsps and their client proteins is crucial for elucidating their functional mechanisms and therapeutic targeting in parasitic diseases. Despite efforts to understand the stoichiometry of chaperone-substrate dynamics, there are still questions that remain to be answered, such as the following: How many Hsps bind simultaneously to a single unfolded substrate protein? What are the species specific stoichiometries for efficient substrate binding dynamics in parasites? What is known under normal physiological conditions is that Hsp70 levels are often in a molar excess relative to their substrates, ensuring efficient capture and refolding of nascent or misfolded polypeptides.^131^ However, during stress conditions in malaria, like other parasites, as unfolded substrate proteins increase dramatically due to heat stress, this results in an upregulation of Hsp70 and its cochaperones.^5,78,130^ It is plausible that when the chaperone-to-substrate ratio falls below a critical threshold, misfolded proteins accumulate, leading to aggregation and cellular toxicity. The effectiveness of Hsps in maintaining proteostasis is therefore not solely dependent on their absolute concentration but rather on the ratio of chaperone to substrate proteins. Cells actively modulate Hsp concentrations to preserve proteostasis through multiple regulatory mechanisms. For instance, the heat shock response (HSR), governed by Apetala 2 (*Pf*AP2), the transcription factor that functions in malaria parasites in place of the eukaryotic heat shock factor 1, enhances Hsp expression during proteotoxic stress, counteracting the increased substrate load.^132^ Furthermore, post-translational modifications, such as phosphorylation, prenylation, and acetylation, fine-tune Hsp activity and substrate affinity, influencing their functional stoichiometry.^133,134^ Additionally, Hsp70-bound substrates damaged beyond refolding repair are frequently targeted for degradation via the ubiquitin-proteasome system (UPS) or autophagy, preventing the accumulation of misfolded proteins and maintaining cellular homeostasis.^135^ Given the essential role of Hsps in parasite survival and stress adaptation, disrupting their stoichiometric balance or regulatory mechanisms represents a compelling strategy for antiparasitic drug development.

### Role of Parasite Hsps in Therapeutics and Diagnosis

Given their critical roles in parasite survival, drug resistance, and pathogenesis, Hsps are emerging as promising therapeutic targets for antiparasitic drug development.^59,136–138^ However, no Hsp-targeting drugs have yet advanced to clinical trials for parasitic infections. In contrast, Hsp90 inhibitors have already been tested in clinical trials for cancer therapy, demonstrating the feasibility of targeting these molecular chaperones for therapeutic intervention.^139^ Notably, parasite-specific Hsp70s have been shown to exhibit distinct structural and functional differences from their human counterparts, presenting a unique opportunity for selective inhibition that could maximize efficacy while minimizing host toxicity.^124,140^ By exploiting the unique structural and biochemical features of protozoan Hsps, targeted drug design could pave the way for next-generation antiprotozoal therapies that circumvent resistance and enhance treatment outcomes (Figure 2). Additionally, combination therapies utilizing Hsp inhibitors alongside existing antiparasitic drugs could enhance treatment efficacy, mitigate resistance, and improve parasite clearance.

**Figure 2:**
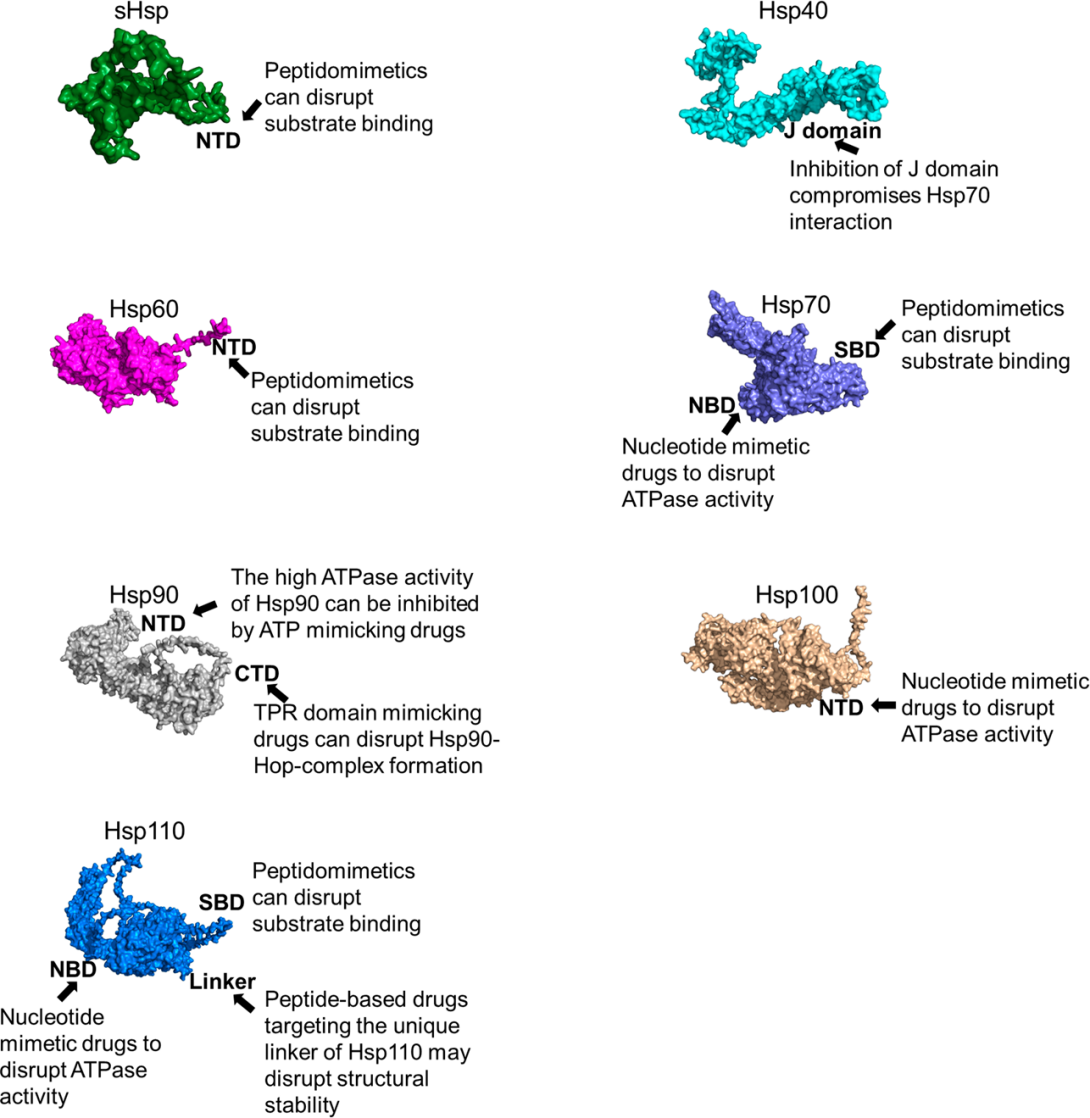
Strategies for Selective Inhibition of Parasite Hsps. Targeting parasite Hsps for selective inhibition presents significant challenges due to their ubiquity and conservation across species. However, structural and functional analyses have revealed species-specific variations, likely arising from evolutionary adaptations that can be exploited for therapeutic intervention. The sHsps and Hsp40, chaperones that lack intrinsic ATPase activity, are most effectively inhibited by using peptidomimetics. In contrast, ATP-dependent chaperones, such as Hsp70 and Hsp900, can be targeted through three distinct mechanisms: (i) inhibition of the ATP-binding site using ATP mimetics, (ii) blockade of the substrate binding domain via peptide mimetics, or (iii) disruption of critical protein–protein interaction motifs essential for ATPase activity modulation. The domains NTD represent N-terminal domain, CTD-C-terminal domain, SBD, substrate binding domain, and NBD nucleotide-binding domain.

Previous research efforts in antiplasmodial inhibitor development have focused on the essential *Pf*Hsp70 and *Pf*Hsp90 chaperone systems. In previous *in vitro* studies, antimicrobial agents including polymyxin-B, (−)-Epigallocatechin-3-gallate, malonganenone A, and phenylthynesulfonamide (PES) have been demonstrated to disrupt *Pf*Hsp70–1 associations with cochaperones.^124,141–143^ *Pf*Hsp90 plays a crucial role in the parasite’s ability to withstand antimalarial drug pressure, making it a compelling target for therapeutic intervention. Recent evidence has also increasingly demonstrated the potential of PfHsp90 inhibitors in compromising parasite viability by disrupting growth and development.^16,144,145^ While the ATP-binding domain of Hsp90 is highly conserved between P. falciparumand humans, recent studies have revealed that subtle structural and conformational differences, especially in the lid dynamics of its nucleotide-binding N-terminal domain (NTD), can be exploited to achieve species-selective inhibition.^145,146^ Structural modeling indicated that altered dynamics near the LXXGA/IXXSG motif are major components of a selective scaffold in *Pf*Hsp90s N-terminal lid.^145^ The compound 7-azaindole’s binding was observed when PfHsp90s' lid adopted a more open conformation, in contrast to closed-lid states stabilized by salt bridges in the human ortholog.^145^ This conformation-specific flexibility appears to enable selective accommodation of the inhibitor scaffold, a dynamic property not mirrored in human Hsp90. It is therefore plausible that multiple regions and conformational states of PfHsp90 contribute to scaffold-specific selectivity, offering complementary paths for the rational inhibitor design.

Beyond their potential as drug targets, plasmodial Hsps are highly expressed during malaria infection and hold promise as biomarkers for disease diagnosis and progression monitoring.^147^ Plasmodial Hsp70 and Hsp90 expression levels are upregulated during febrile episodes and are involved in host–pathogen interaction in immunomodulation, therefore may correlate with malaria severity, making them valuable indicators of infection status.^14,24,148–150^ A latex particle agglutination assay utilizing antibodies against P. falciparumHsp70 achieved 84% sensitivity and 90% specificity for parasite detection.^151^ Similarly, an ELISA-based assay using recombinant P. vivaxHsp70 (*Pv*Hsp70) to detect anti-*Pv*Hsp70 antibodies showed high sensitivity (88.8%), effectively detecting antibodies from both P. falciparumand P. vivaxpatients.^152^ Despite Hsps being generally conserved, several studies indicate that infection-specific immune responses can be mounted against parasite-derived Hsps. The immunogenicity of Hsp90 during Plasmodium yoelii infection using both human and murine models was demonstrated.^147^ In that study, patients and murine models infected with P. yoelii had elevated serum levels of anti-Hsp90 antibodies, suggesting that the unique features of parasite Hsp90 may enable selective immune recognition. Another study examining individuals living in a malaria-endemic region of West Africa displayed a significant antibody response to native *Pf*Hsp70–1 antigen, with 70% of adults having an immune response.^153^ This highlights the presence of parasite-specific epitopes within plasmodial Hsp70 that are immunologically recognized in infected populations. These findings collectively support the potential of Plasmodium Hsps as diagnostic biomarkers, particularly for early malaria detection and monitoring of disease progression. Further optimization of Hsp-based diagnostic platforms could enhance their clinical utility, offering cost-effective and accessible malaria detection strategies in endemic regions.

In T. gondii, Hsps are crucial for the tachyzoite-to-bradyzoite transition, a key survival mechanism. *Tg*Hsp90 and *Tg*Hsp70 regulate parasite replication and stress adaptation in host cells, making them attractive targets for the development of antitoxoplasmal drugs.^61,154^ Several studies have investigated *Tg*Hsp90 inhibition as a strategy to block parasite growth and differentiation. Notably, a previous study demonstrated that geldanamycin, a benzoquinone ansamycin antibiotic, effectively inhibited the conversion of T. gondiicells between tachyzoite and bradyzoite stages, preventing both tachyzoite-to-bradyzoite differentiation and the reverse transition.^18^ Given its essential role in T. gondiisurvival and pathogenesis, targeting *Tg*Hsp90 with small-molecule inhibitors could provide a promising therapeutic approach by modulating stress-induced signaling pathways and protein homeostasis. While *in silico* studies have explored the potential inhibition of other Hsp classes, such as Hsp70 and Hsp60,^137^ experimental validation through in vitro assessments remains limited. A previous study demonstrated that anti-Hsp70.1 antibodies, in combination with a logistic probability test, can effectively confirm clinically suspected cases of ocular toxoplasmosis, providing a potential diagnostic marker for disease identification.^155^ Moreover, Hsp-based serological tests have been shown to detect latent infections and differentiate active from dormant Toxoplasma stages,^155^ further supporting the diagnostic potential of Hsp biomarkers. These findings suggest that Hsps could be leveraged for the development of novel point-of-care (POC) diagnostic tools, enhancing early detection and disease monitoring in toxoplasmosis.

Similarly, Trypanosoma species, including T. bruceiand T. cruzi, rely on Hsps for life cycle progression and adaptation to host environments. Hsp70 and Hsp90 from *Trypanosomal sp* are essential for survival, differentiation, and antigenic variation. Several studies have explored the inhibitory effects of the Hsp90 inhibitors on different trypanosomal species proliferation.^21,130^ A study by Meyer and Shapiro,^63^ demonstrated that the geldanamycin derivatives 17-AAG and 17-DMAG exhibited high selectivity against T. brucei. Interestingly, both oral and parenteral administration of 17-DMAG successfully cured mice of a normally lethal T. bruceiinfection, highlighting its potential as a viable treatment option.^63^ These findings provide strong evidence supporting the use of Hsp90 inhibitors to study trypanosome biology and their potential clinical development for treating trypanosomiasis. Although there are limited data on trypanosomal Hsp70 inhibitors, Hsp70 ATPase inhibitors could interfere with chaperone-assisted protein folding, leading to trypanosome cell death. Beyond their therapeutic potential, trypanosomal Hsps may also serve as biomarkers for the development of novel point-of-care diagnostics. However, data on Hsp-based biomarkers for early disease detection remain scarce, necessitating further studies to explore their diagnostic utility in trypanosomiasis.

Ongoing research has significantly contributed to the design and evaluation of Hsp inhibitors as potential antileishmanial agents, with several inhibitors targeting leishmanial Hsps being tested.^138,156^ However, despite promising *in vitro* findings, challenges remain in translating these inhibitors into clinically effective therapies for leishmaniasis. A number of studies have demonstrated that Hsp90 inhibitors from *Leishmania sp* can block parasite growth and stress adaptation.^157,158^ Additionally, inhibitors targeting L. donovaniHsp78 have shown promising antileishmanial activity, further expanding the potential of Hsp-targeting therapies for leishmaniasis.^159^ Given the essential role of Hsp78 in proteostasis and stress adaptation, its inhibition disrupts parasite survival and virulence, making it a compelling target for therapeutic intervention. However, further studies are needed to develop efficacious inhibitors with translational potential for the treatment of leishmaniasis. Combination therapy of promising Hsp inhibitors with standard treatments should also be explored for enhanced treatment efficacy. Hsp70 has previously been identified as a dominant antigen in leishmania infections, with studies revealing that anti-Hsp70 antibodies were detected in 95% of sera from mucocutaneous (ML) and localized cutaneous (CL) patients.^160^ Beyond Hsp70, L. infantumHsp90 has also shown potential as a highly specific and sensitive biomarker for serodiagnosis, with minimal cross-reactivity with other infectious diseases.^161^ The development of Hsp-based biomarkers for point-of-care diagnostics could significantly enhance the early detection and disease monitoring in leishmaniasis. Integrating these biomarkers into serological assays such as ELISA, lateral flow tests, or nanoparticle-based platforms may improve accuracy, accessibility, and cost-effectiveness, particularly in endemic regions. However, further clinical validation and large-scale studies are necessary to standardize Hsp-based diagnostic approaches for routine clinical use.

## Conclusions

Parasite heat shock proteins (Hsps) exhibit distinct structural and biochemical properties that are crucial for protein homeostasis and survival within the host environment. These unique features present potential vulnerabilities that can be strategically exploited for selective therapeutic targeting, ultimately leading to parasite cell death. While significant research efforts have primarily focused on Hsp90 and Hsp70 as drug and diagnostic targets, a broader, systems-level approach to the entire Hsp network could accelerate the discovery of novel therapeutic strategies (Figure 3). Further studies are essential to elucidate the molecular intricacies of parasite Hsps, which could pave the way for the development of highly specific and effective antiparasitic interventions.

**Figure 3:**
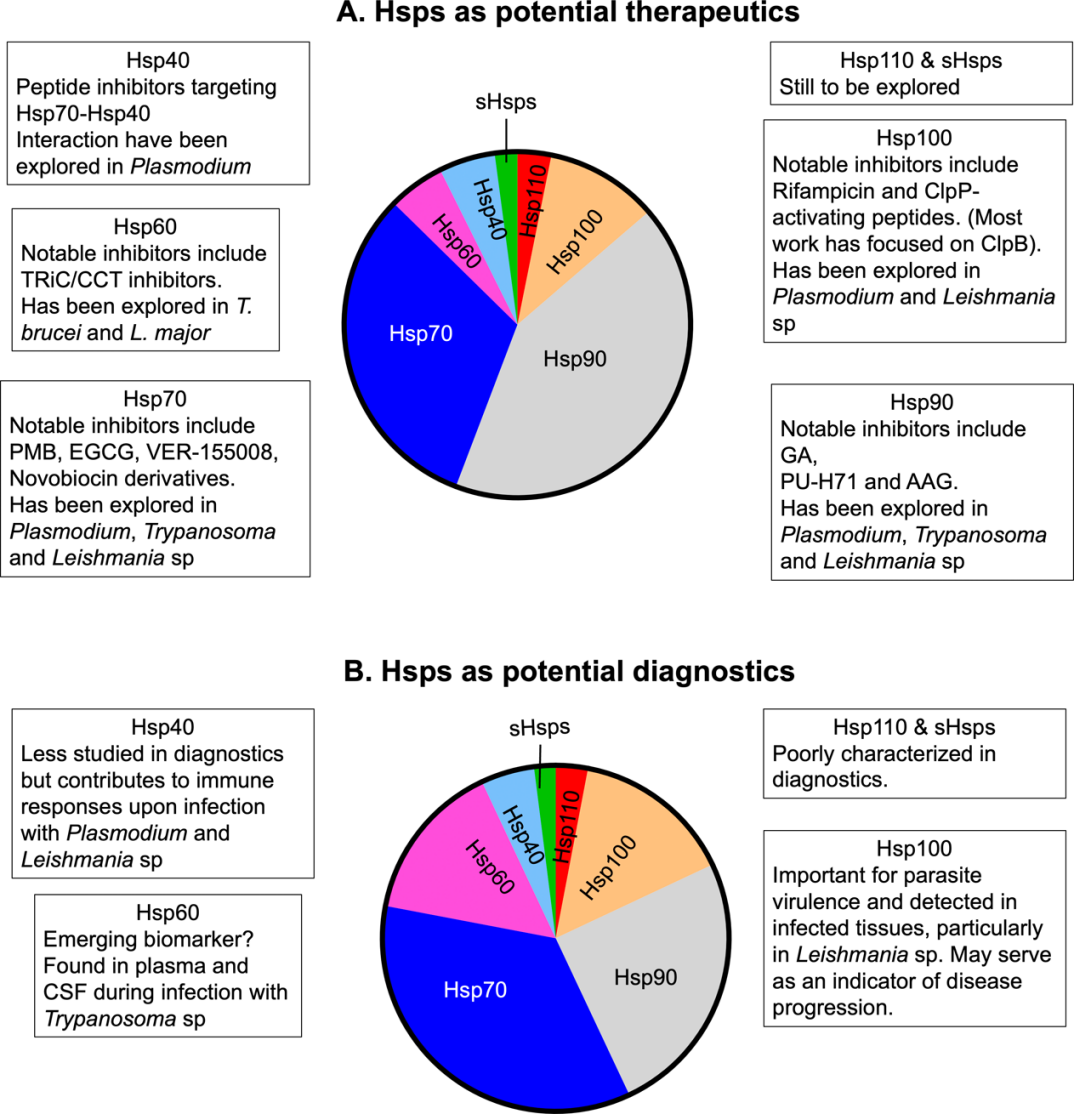
Disproportionate research focus on parasite Hsps for Therapeutic and Diagnostic Applications. The distribution of research efforts on parasite Hsps reveals a strong bias toward Hsp90 and Hsp70, which dominate studies related to drug discovery (A) and diagnostic marker development (B). This preferential focus is likely driven by the well-characterized ATP-binding sites of these chaperones, making them more accessible targets for inhibition. In contrast, despite the crucial role of Hsp40 in protein homeostasis and its expanded family in parasites, it remains significantly underexplored as a potential therapeutic or diagnostic target. A more balanced research approach, incorporating the broader Hsp network, could unlock new opportunities for parasite-specific interventions.
